# Motivation of emergency medical services volunteers: a study of organized Good Samaritans

**DOI:** 10.1186/s13584-020-00370-9

**Published:** 2020-06-02

**Authors:** Michael Khalemsky, David G. Schwartz, Raphael Herbst, Eli Jaffe

**Affiliations:** 1 Graduate School of Business Administration, Bar-Ilan University, Ramat Gan, Israel; 2 Magen David Adom, Tel Aviv, Israel

**Keywords:** Volunteer, Good Samaritans, Emergency services, Motivation

## Abstract

**Background:**

Early professional care in emergencies is beneficial in general and its utility has been proven in many studies, particularly in regard to out-of-hospital cardiopulmonary arrest. A person in distress can expect help from two sources: bystanders, including family members, community members, and complete strangers; and professionals, including emergency medical services, first responders, firefighters, and police officers. Emergency Medical Services try to achieve faster first response times through various approaches. Recent technological and social developments have enabled a new form of Emergency Medical Services volunteering, called Organized Good Samaritans, which represents a new layer between occasional volunteers and time-donation volunteers. Organized Good Samaritans are people with a medical background, particularly off-duty medical professionals who are willing and able to provide first aid in emergencies in their vicinity.

**Methods:**

A qualitative formalization of technology-enabled Organized Good Samaritans is presented. One thousand eight hundred Israeli National Emergency Medical Services volunteers were surveyed using Clary and Snyder’s Volunteer Functions Inventory instrument. Demographics, professional backgrounds, and volunteering functions of Time-Donation Volunteers and Organized Good Samaritans are compared.

**Results:**

Significant differences between Organized Good Samaritans and Time Donation Volunteers were found. Demographically, Organized Good Samaritans are older and the percentage of males is higher. Professionally, the percentage of physicians and nurses among Organized Good Samaritans is higher. Motivation measures find that the motivation of Organized Good Samaritans is higher and the order of importance of the volunteering functions differs.

**Conclusion:**

A clearly identifiable and differently motivated class of emergency services volunteers has emerged. An appropriate information technology infrastructure enables Emergency Medical Services organizations to integrate Organized Good Samaritans into core business processes to shorten response times to emergencies**.** Organized Good Samaritans provide a volunteering opportunity for highly skilled people unable to be Time-Donation Volunteers. Our findings provide an empirical basis for further research on Organized Good Samaritans integration into Emergency Medical Services operations. Emergency Medical Services administrators can use these findings to establish an Organized Good Samaritans infrastructure and adjust recruitment and retention. This study is limited to one national Emergency Medical Services organization in Israel. Cultural differences can impact results in other countries. Organized Good Samaritans effectiveness should also be studied in terms of response times and medical outcomes.

## Background

A person in distress can expect help from two sources: bystanders, including family members, community members, and complete strangers; and professionals, including emergency medical services, first responders, firefighters, and police officers.

Early professional care in emergencies is beneficial in general and its utility has been proven in many studies, particularly in regard to out-of-hospital cardiopulmonary arrest (OHCA) [1–3]. Emergency Medical Services (EMS) organizations and health policy makers try to achieve faster response times through various approaches, but time remains a significant factor. Although there is no universally accepted standard for EMS response times, the most widely used goal is to respond to 90% of calls within 9 min in urban areas and within 15 min in rural areas [4]. The impact of elapsed time is significant. For example, in cases of OHCA, the survival rate decreases by 10% for every minute of delay in initiating cardiopulmonary resuscitation (CPR) [5]. People in distress often must rely on Good Samaritan response until ambulance arrival. For example, one 2013 study shows that bystanders performed CPR in 40.1% of OHCA events in the US [5].

Until only recently, a Good Samaritan had to eye-witness an emergency or hear a cry for help in order to act. A medical doctor, even one who volunteers regularly for a local EMS or fire department, could be sitting in a cafe unaware that someone needs critical care 100 m away.

Technological advances and the penetration of smartphones provide a new opportunity as two-thirds of Americans [6] and 74% of Israelis [7] now own a smartphone. We can now organize Good Samaritans, record their knowledge and skills, track their location, and inform them about emergency events in their vicinity according to their abilities and responder profile.

For this research the following definition of *Organized Good Samaritan*s (OGS) is adopted: *Organized Good Samaritans are occasional volunteers managed and called to action through smartphone apps, when mediated by an organization that holds primary responsibility for responding to the emergency event*.

### Volunteers in emergency medical services

Volunteers form a significant part of the personnel of many EMS organizations [8–10]. In the US, the urban EMS workforce is comprised of approximately 30% volunteers, with rural EMS workforces reaching up to 75% volunteer staffing [8, 10]. In Austria volunteers make up more than 80% of EMS personnel [11] and in Israel about 89% of the National EMS (Magen David Adom or MDA) personnel are time-donation volunteers.

The classic form of volunteering in EMS is by *time donation*, such as one shift per week [10, 12]. EMS organizations experience significant shortages of *time-donation volunteers* (TDV), especially in rural areas [8, 10]. Significant numbers of volunteer-based organizations seek to leverage information and communication technologies to enhance administrative and operational efficiency and effectiveness [13].

### Efforts to achieve faster first aid

EMS organizations and health policy makers try to achieve faster response times through various approaches. These include Automatic External Defibrillator (AED) deployment in public places [14–17]; the use of drones to deliver emergency equipment [18]; and the establishment of different networks of first responders [19–28]. The density of first responders in a given geographic area is strongly correlated with faster first aid [29].

### Good Samaritan response by medical professionals

People help each other in emergency situations and the phenomenon of “bystander intervention” has been vigorously studied over the past five decades [30–34]. Off-duty doctors and other medical professionals have the highest potential to help given their medical knowledge and training, and have been identified as an important source of volunteers in disaster recovery plans [35]. About 80% of physicians have had an opportunity to provide a Good Samaritan response and 93% agreed to provide help when asked [36–38]. In 1959 California gave physicians immunity from civil liability resulting from provision of a Good Samaritan response and since then all other states have followed suit [39].

### Smartphone-based networks of volunteers

mHealth is defined as “*healthcare to anyone, anytime and anywhere by removing temporal and locational constraints*” [40]. Developments in mHealth have led to a sharp increase in the availability of applications (apps) supporting medically motivated physical interaction between app users, leading to potentially significant changes in healthcare delivery and the structure of emergency response organizations [41, 42]. Multiple apps exist in this area, such as Pulsepoint [43], AllergyHero [44], and UnityPhilly [45]. An extensive review of emergency response apps can be found in the study by Gaziel-Yablowitz and Schwartz [46].

Most such apps are unmediated by EMS call centers, enabling people in distress to directly ask other app users for help, and potential responders react based on the app information provided. The requester must have the same app as the responder. Mediation by an emergency organization eliminates the aforementioned limitation. EMS mediation also means that the requester does not need to rely on an app, but can resort to the tried and true emergency phone call to 911 (101 in Israel). With EMS mediation of app communications, the command and control center of the emergency service can dispatch OGS to any event, regardless of how it was reported.

Mobilization of off-duty police officers, military personnel, or medical professionals in emergency situations existed long before the smartphone revolution, by means of pagers, two-way radios, and telephones. However, an organization that wants to integrate OGS into its EMS processes needs a completely different technological approach in order to effectively locate OGS who are close to the scene, notify them, and process their feedback about their ability to respond. The “Life Guardians” system developed by MDA [47] includes an app and a server that tracks the location of OGS and dispatches them automatically or at the discretion of a dispatcher. This system has managed thousands of OGS since its introduction in 2016.

### Motivation and willingness to volunteer

The willingness to volunteer has been studied from several perspectives. Individual characteristics, such as age, social background, resources, and health status, help predict an individual’s voluntary action participation [48–50]. Motives such as altruism, self-actualization, need for power, need for mutual support, and self-esteem explain many aspects of volunteer behavior [51–57]. Organizational factors affecting volunteers such as an organization’s values and attitudes have also been studied [58].

Understanding the different characteristics and volunteering functions of volunteers can help volunteer-dependent organizations focus their recruitment efforts on the most relevant people and develop programs that help satisfy the volunteers’ motives [59–63].

Previous studies have identified several motives for volunteering as a first responder, including altruism, need for social interaction, improvement of skills and knowledge, getting experience for future careers, and self-esteem [64]. Having a flexible schedule and controlling the decision of when to be on duty were found to be important factors for many volunteers [9, 64].

Motivation and satisfaction can vary between organized volunteers such as TDV and occasional volunteers such as Good Samaritans [55].

Clary and Snyder identified six motivational functions served by volunteering [52]:

1. Values – volunteering is based on important values like humanitarianism.
2. Understanding – volunteering in order to learn more or to exercise unused skills.
3. Enhancement – volunteering in order to grow and develop psychologically.
4. Career – volunteering in order to get career-related experience.
5. Social – volunteering in order to strengthen social relationships.
6. Protective – volunteering in order to reduce negative feelings or problems.

Clary and Snyder [52] developed a widely accepted and validated research tool, the Volunteer Functions Inventory (VFI), which enables assessment of these six volunteering functions.

### Organized good Samaritans – MDA Life Guardians

Magen David Adom (MDA) is the Israeli national EMS, belongs to the International Red Cross and Red Crescent Movement, and serves as the Israeli Red Cross National Society. MDA has operated a nationwide volunteer first responder (VFR) network since 2000. In 2016 MDA began to integrate the OGS approach into its organizational processes. MDA launched a new program, *Life Guardians*, through which people with medical certification or training are invited to download a smartphone app, making them identifiable and reachable based on geographic proximity to an emergency event, and enabling them to provide a first response. The identity and medical certifications of *Life Guardians* are verified. Those approved are provided basic equipment and supplies by the EMS. Upon emergency event verification, the event’s characteristics, the volunteers’ capabilities, and the expected arrival time of the ambulance and VFR are taken into the account [47, 65]. MDA tries not to overload Life Guardians, which may lead to burnout, and dispatches them only to high-priority events such as resuscitation, unconsciousness, choking, and severe trauma, when the expected time of arrival of an ambulance or a VFR is longer than the expected response time of OGS. The *Life Guardians* project differs from the aforementioned networks of volunteers [19–28] in that it is not condition-specific (other projects usually focus on a single condition, mostly OHCA). MDA requires and records existing medical background and doesn’t provide any training. Similar to the *Life Guardians* program, Australia’s Rural Emergency Responder Network (RERN) acts on a smaller scale by targeting the participation of general practitioners in rural areas [66].

## Methods

### Research model

The study provides a qualitative formalization of technology-enabled OGS. This enables the formal analysis of the phenomenon whereby organizations leverage technology to integrate occasional volunteers into the network of first responders to emergency situations. A brief outline of the required technology is also presented.

The qualitative analysis is followed by a quantitative comparison of the professional background, demographics, and volunteering functions of traditional time-donation EMS volunteers versus those of OGS.

### Data collection

The sample for this study consisted of volunteers from a national EMS organization in Israel, Magen David Adom. It has an employed professional workforce of 2200 medical personnel, augmented by 20,000 certified time-donation volunteers and 17,000 certified OGS volunteers.

For this research a random representative sample of 800 TDV and 1000 OGS within MDA were surveyed. 331 (41%) valid responses were received from the time-donation volunteers and 601 (60%) valid responses were received from the OGS.

### Research techniques

Clary and Snyder’s VFI instrument [52] was used to assess the volunteering functions of the two types of volunteers. Data was collected using Google Docs between March and May 2017. The VFI instrument consists of 30 statements, ranked on a 7-point Likert scale with responses ranging from 1: not at all important/accurate to 7: extremely important/accurate. Each volunteering function was scored based on five distinct statements in the instrument. The complete questionnaire is presented in Appendix.

For each response, the six motivational functions were calculated.

To assess the questionnaire’s reliability, Cronbach’s alpha analysis was used, with results indicating that the questionnaire is reliable (Table 1).

**Table 1:** Cronbach’s alpha values

Function	Time donation	OGS
Values	0.836	0.767
Understanding	0.885	0.876
Enhancement	0.859	0.840
Career	0.889	0.906
Social	0.698	0.753
Protective	0.821	0.801

## Results

### Demographic data

Table 2 presents the gender and age statistics of the different types of volunteers.

**Table 2:** Gender and age

Type		Male	Female	Not reported	All
Time donation	Gender	152 (45.92%)	177 (53.47%)	2 (0.6%)	331 (100%)
	Average age^a^	25.33 (9.8)	23.44 (8.99)	49 (0)	24.4 (9.51)
OGS	Gender	452 (75.21%)	142 (23.63%)	7 (1.17%)	601 (100%)
	Average age^a^	38.32 (11.95)	39.4 (12.48)	56 (0)	38.61 (12.1)
All	Gender	604 (64.81%)	319 (34.23%)	9 (0.97%)	932 (100%)
	Average age^a^	35.68 (12.84)	31.18 (13.38)	41.67 (15.58)	34.27 (13.2)

^a^Standard deviation is reported in parentheses

A Z-test for differences in proportions revealed that the percentage of males is significantly (*p*-value = 0.000) higher in OGS (75.21%) than in TDV (45.92%).

A t-test for independent samples showed that OGS are significantly (*p* = 0.000) older (mean = 38.61 years) than TDV (mean = 24.4 years). The difference between the average age in the two samples is 14.21 years.

### Professional backgrounds

**Fig. 1:**
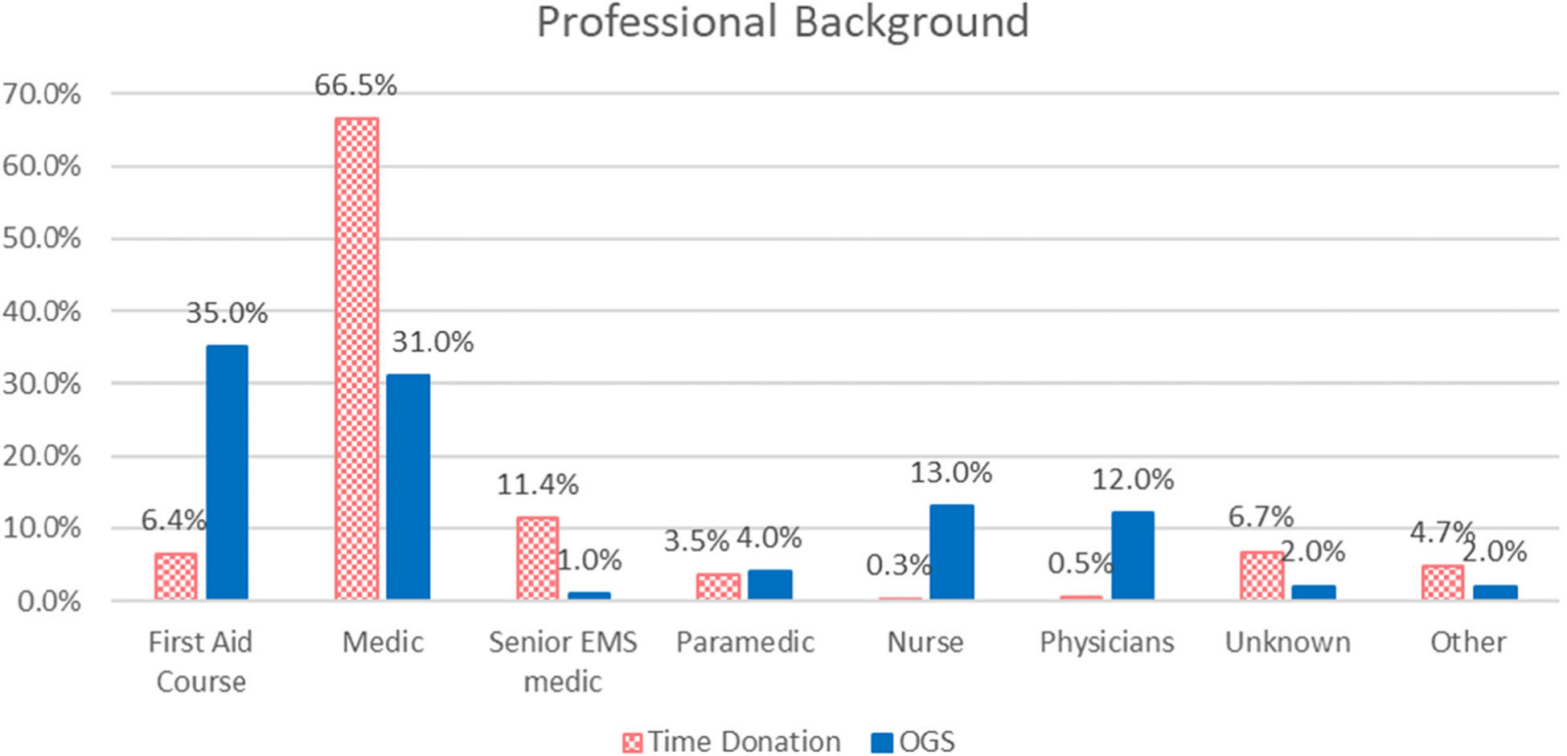
Professional background of the life guardians compared to time-donation volunteers

Figure 1 compares the professional background of OGS and TDV.

The *χ*^2^ test for independence showed the professional background of OGS and TDV to be significantly (*p*-value = 0.000) different. The proportion of physicians and nurses is much higher among OGS (12 and 13%, respectively) than among TDV (0.5 and 0.3%, respectively).

### Differences in volunteering functions

**Fig. 2:**
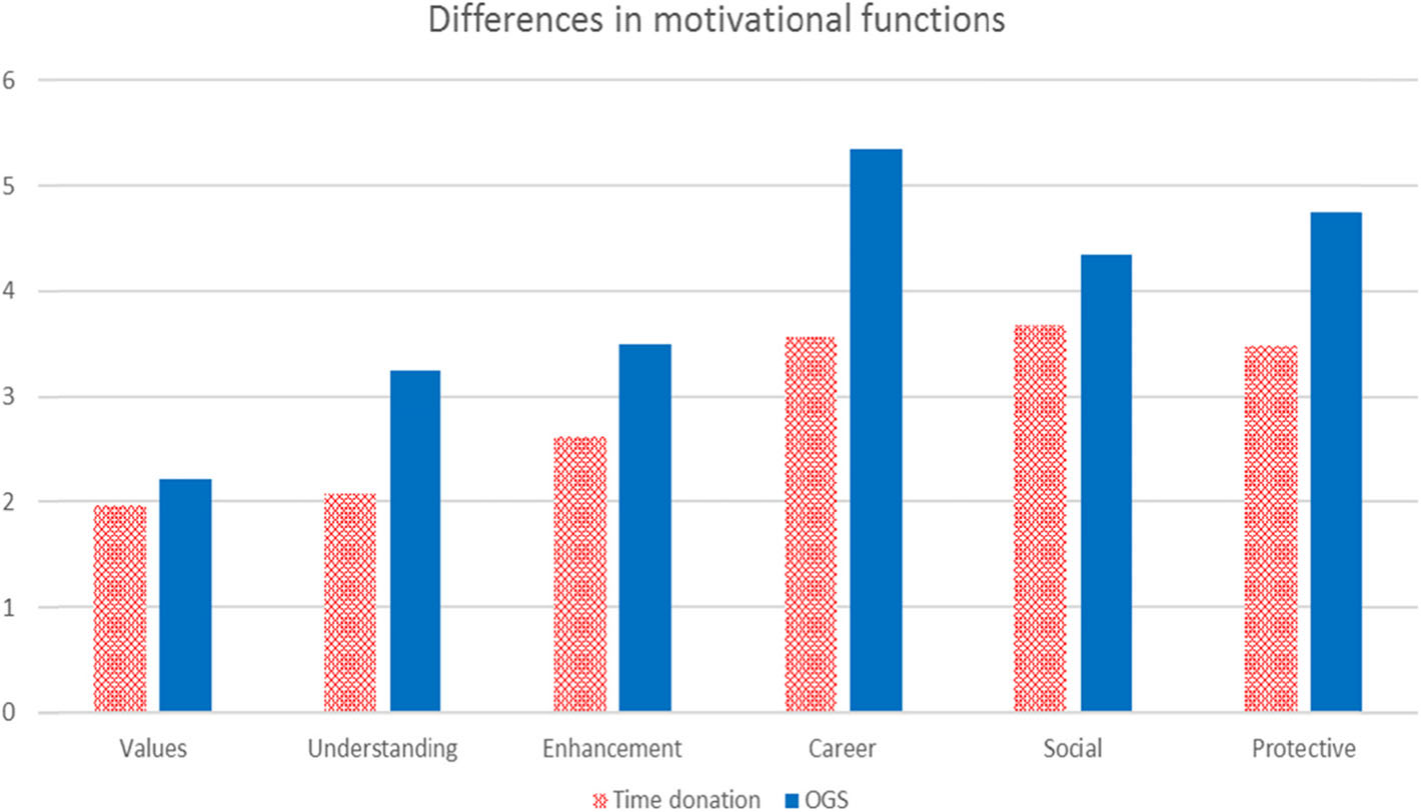
Differences in motivational functions

Figure 2 shows the differences in volunteering functions between TDV and OGS on a 7-point scale ranging from 1 (not at all important) to 7 (extremely important).

All six volunteering functions are higher for OGS than for the TDV. A t-test for independent samples was performed. The *p*-value for all functions was lower than 0.05, indicating that the differences in volunteering functions between the two groups are significant.

Table 3 presents a comparison of the motivational functions.

**Table 3:** Differences in motivational functions between TDV and OGS

	Values	Understanding	Enhancement	Career	Social	Protective	Average
TDV	1.96	2.07	2.62	3.57	3.67	3.49	2.90
OGS	2.21	3.24	3.50	5.35	4.35	4.75	3.40
Difference (points)	0.24	1.17	0.88	1.78	0.68	1.26	1

Table 4 presents the differences in the ranked importance of the volunteering functions.

**Table 4:** Differences in the ranked importance of volunteering functions

TDV	OGS
Social (3.67)	Career (5.35)
Career (3.57)	Protective (4.75)
Protective (3.49)	Social (4.35)
Enhancement (2.62)	Enhancement (3.50)
Understanding (2.07)	Understanding (3.24)
Values (1.97)	Values (2.21)

### Moderation

#### Gender and age

When the comparison was performed separately for males and females, the results were only slightly different. Two separate t*-*tests for independent samples were performed: one for males and one for females. For males, the differences in all functions except *values* are significant while for females the differences in all functions are significant.

Since there are significant differences in the average age of TDV and OGS, the matched-pairs technique [67] was used to match records by age and gender and perform paired-samples t-tests. Results suggest that there is no moderation by age and gender and that the differences in all volunteering functions except *values* are significant.

#### Length of service as a volunteer

The *Life Guardians* OGS project was launched in 2016 whereas many TDV have served for 10 years or more.

In order to check that length of service as a volunteer didn’t affect our results, a t-test was performed comparing OGS with TDV with up to 2 years of experience. For all six variables, Levene’s test of equality of variances provided a *p*-value > 0.05, meaning that in all t-tests the equality of variances can be assumed. The results of independent samples t-tests for each function are: *values* (*t* = 3.187, *p*-value = 0.001), *understanding* (*t* = 8.756, *p*-value = 0.000), *improvement* (*t* = 6.901, *p*-value = 0.000), *career* (*t* = 12.361, *p*-value = 0.000), *social* (*t* = 4.833, *p*-value = 0.000), *protective* (*t* = 11.091, *p*-value = 0.000). Accordingly, length of service as a volunteer doesn’t affect the results and the differences remain significant.

### Multivariate analysis of the motivational differences between TDV and OGS

A principal component analysis revealed two possible components with high correlations between the variables and the component, and thus no variables were omitted. A binary logistic regression with a dependent variable representing the type of volunteering (OGS vs. TDV) and independent variables, including age, gender, and the six volunteering functions resulted in a significant model (*p*-value = 0.000) where the Cox and Snell pseudo *R*^*2*^ = 0.375 and the Nagelkerke pseudo *R*^*2*^ = 0.519. Table 5 presents the results of the regression analyses.

**Table 5:** Results of the binary logistic regression

Variable	*β* (estimate)^a^	95% CI	Wald χ2	*P*
Age	−0.104	−0.126 to − 0.082	86.316	0.000
Gender	1.312^b^	0.919 to 1.706	42.813	0.000
Values	0.263	0.033 to 0.492	5.023	0.025
Understanding	−0.578	−0.824 to − 0.333	21.407	0.000
Enhancement	0.404	0.17 to 0.639	11.429	0.001
Social	−0.238	−0.423 to − 0.054	6.438	0.011
Protective	−0.16	−0.336 to 0.017	3.13	0.077
Career	−0.114	−0.269 to 0.041	2.081	0.149

^a^Positive *β* values are associated with a higher probability of becoming a TDV and negative *β* values are associated with a higher probability become becoming an OGS

^b^Males had a higher probability of becoming an OGS than a TDV

The correlation matrix did not show any strong correlations between independent variables, and thus no multicollinearity is suspected.

### J48 classification tree analysis

**Fig. 3:**
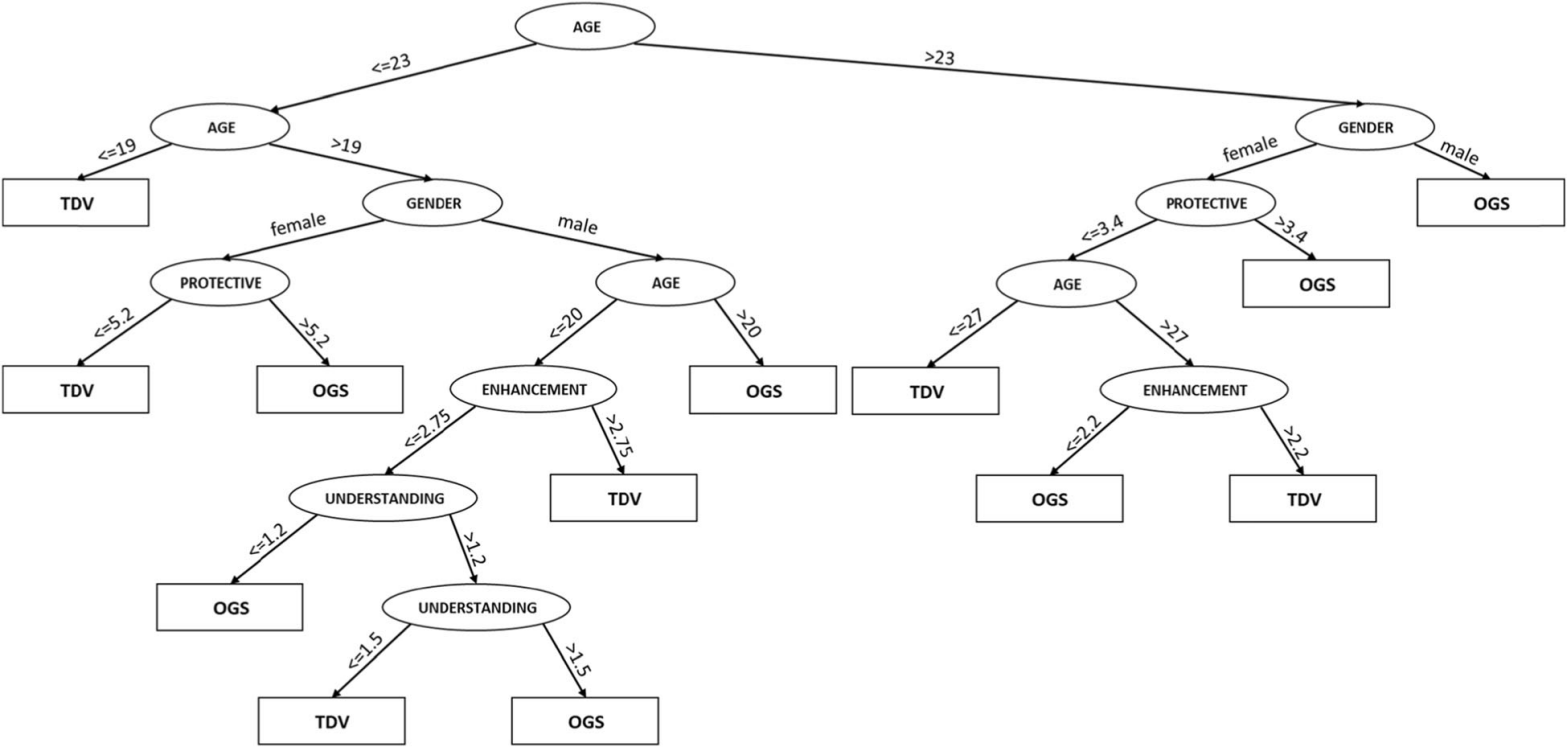
J48 classification tree for TDV vs. OGS

In order to analyze the factors that influence one’s decision to become a TDV or an OGS, the J48 classification tree analysis [68] was performed. The tree correctly classified 78.9% of the cases, as shown in Fig. 3.

### Transition of volunteers between TDV and OGS

80% of OGS surveyed expressed willingness to become time-donation volunteers. Results also show that 28.3% of OGS were former TDV in MDA (MDA doesn’t allow active TDV to concurrently serve as OGS).

## Discussion

### Organized good Samaritans

**Fig. 4:**
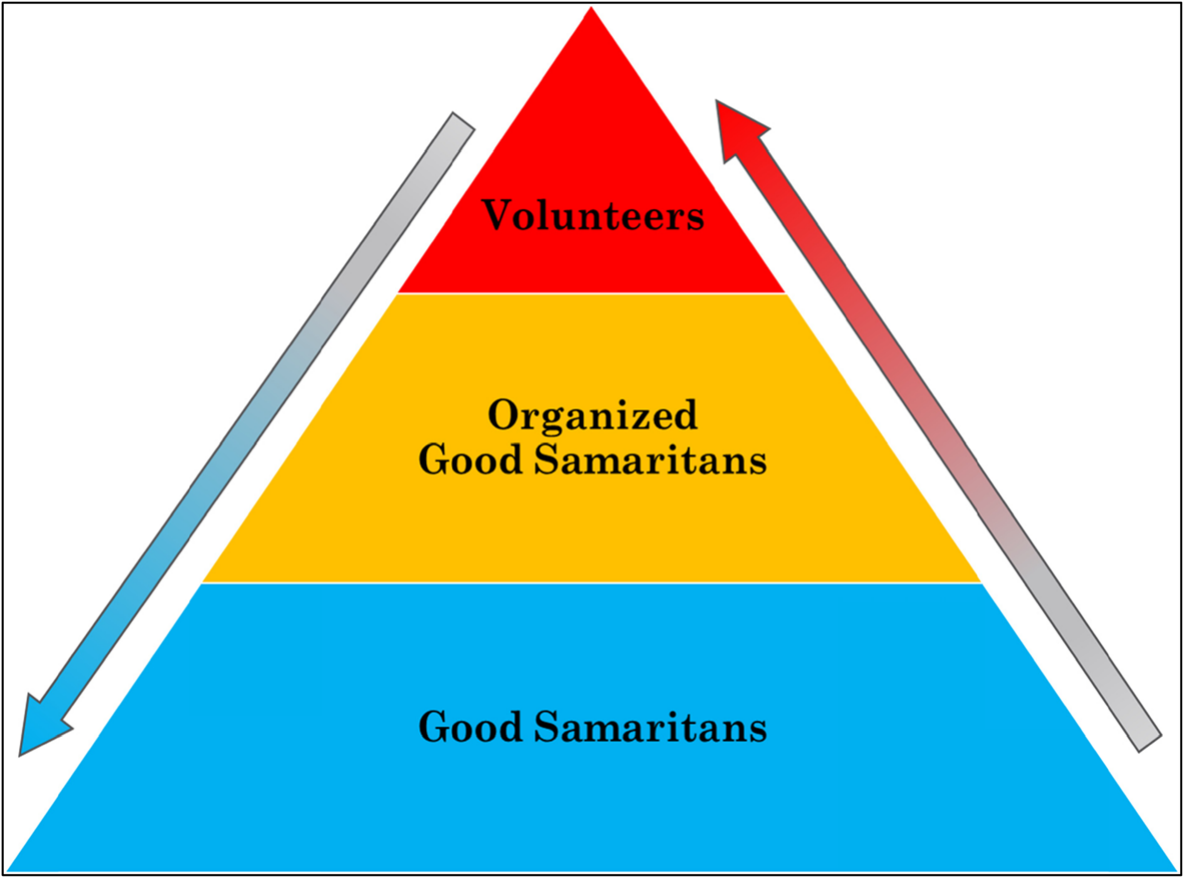
Volunteers taxonomy

It is helpful to characterize OGS within a broader taxonomy of volunteers, as shown in Fig. 4. The time-donation volunteers are the most organized part of the pyramid. They are an integral part of the EMS personnel, wear a uniform, must successfully complete courses and certifications offered by EMS, and can be promoted within the EMS organization. The Good Samaritans at the base of the pyramid are occasional volunteers: any layperson who witnesses an emergency can, and in many countries is obligated to, provide help to a person in distress. Organized Good Samaritans are a hybrid of the aforementioned two types of volunteers. On the one hand, they do not have scheduled shifts to attend or a uniform to wear, but on the other hand, they are not fully occasional: by joining the program, they greatly increase their chances of involvement in the organization’s life-saving activities.

Another important difference is that Organized Good Samaritans are vetted and only people that meet EMS requirements can join the program. The managing organization can provide its OGS with some training and equipment or supplies. For example, the MDA provides the *Life Guardians* with basic equipment, supplies, and a certificate that allows access to emergency scenes in the event that it becomes a restricted area.

### Potential transition between volunteer categories

The upward arrow in Fig. 4 represents two potential transitions: occasional Good Samaritans with a medical background who become Organized Good Samaritans in order to increase their chances of involvement in the organization’s life-saving activities; and Organized Good Samaritans who become time-donation volunteers. As we already mentioned, in this survey, 80% of the OGS expressed willingness to become time-donation volunteers.

The downward arrow in Fig. 4 represents two potential transitions: Organized Good Samaritans who return to the status of occasional Good Samaritans because they are not interested in continuing with the program; and time-donation volunteers who transition to OGS because they are not able to continue to donate time on a regular daily, weekly, or monthly schedule, but still want to volunteer albeit in a less binding framework. As previously mentioned, our sample showed 28.3% of the OGS were former TDV in MDA.

Clearly, TDV and OGS may also serve as occasional Good Samaritans when they are off duty and spontaneously encounter an emergency situation in which they can help. Understanding movement between volunteer categories should be the focus of future research.

### Differences in the professional background and demographic characteristics of OGS and TDV

The gender distribution is significantly different between OGS and TDV. This finding is consistent with previous studies that found gender differences in bystander response behavior [69], emergency response [70], OGS response [71] and EMS providers [72]. This can be explained by the fact that while TDV act as a team, OGS have to act alone.

Our finding that TDV are significantly younger than OGS is not surprising because in the case of MDA many TDV are young people, even high school students. OGS are primarily mid-career medical professionals who do not have enough time to donate and are looking for a less binding form of volunteering. This finding differs from previous studies that have found that the average age of emergency services volunteers is increasing, due to sociological changes such as two-income families and longer working hours [10, 73].

We also find that the proportion of physicians and nurses is much higher in OGS than in TDV. Previous studies have found that physicians are half as likely to volunteer as the general public, that they cite lack of time as the primary barrier to volunteering [74], and that full-time employment reduces the probability of volunteering [75]. It seems that the OGS platform provides a volunteering opportunity to those who are not able to serve as TDV.

### Differences in volunteering functions

Significant differences between the volunteering functions of OGS and TDV can be explained by several complementary factors.

Most time-donation volunteers have non-medical careers. By contrast, most OGS are active medical professionals for whom professional achievement or clinical practice might be a more important priority, as noted in previous studies [76, 77]. Related findings were observed by Switzer et al. who compared motivational factors between medical students and the general population of volunteers and found that the importance of *career* was significantly higher among medical students [78].

The high importance of the *social* function for TDV can be explained by the intensive social interaction that TDV experience during their shifts, training, and other activities organized by the EMS. By contrast, OGS do not participate in such social activities or work in shifts during which they would meet other volunteers.

Time-donation volunteers expect emergency events during their shifts. By contrast, OGS perform their daily activities until they are notified of an emergency, and hence their response is spontaneous. Kulik at al. found significant differences in motivational factors between spontaneous and organized volunteers [55].

### Volunteering functions differences and their magnitude

The differences between OGS and TDV in all volunteering functions except *values* are significant. OGS were found to have higher motivation in all six factors. However, the magnitude of these differences varies among the volunteering functions. As can be seen in Table 3, the average difference is 1 point or 34%. The highest (above the average) differences are observed in the *understanding*, *career*, and *protective* functions while the lowest (below the average) differences are observed in the *enhancement*, *social*, and *values* functions (the difference in *values* is not statistically significant). We conclude that overall OGS are more highly motivated than TDV, but the most significant differences are in the *understanding*, *career*, and *protective* functions.

***Values*** shows no significant difference between OGS and TDV. The relatively low importance of this function for both groups of volunteers does not match the results of previous studies [52, 57, 63, 78] and is surprising. Further research is needed to investigate this finding, which lies beyond the scope of the present study.

***Understanding*** is significantly higher in OGS than in TDV. The difference has a high magnitude, 1.17 points or 56%, but its relative importance is low, fifth place for both groups of volunteers. Volunteer satisfaction is influenced by matching activities to motivations [79]. Opportunities to learn and develop new skills are an effective tool for volunteer retention [80]. Thus, the implication of this finding is that provision of nonmandatory training by the EMS to OGS may be useful in OGS recruitment and retention.

***Enhancement*** is significantly higher in OGS than in TDV. However, this function was found to be less important for both groups of volunteers (fourth place) and the magnitude of the difference between the groups is relatively low at 0.88 points or 33%. The implication of this finding is that enhancement-oriented activities by administrators of EMS organizations, such as raising the self-esteem of the volunteers, can be more effective for recruitment and retention of OGS than for TDV.

***Career*** is significantly higher in OGS than in TDV. This function ranked as most important for OGS and second in importance for TDV. The magnitude of the difference between OGS and TDV is high at 1.78 points or 50%. The implication of this finding is that administrators of EMS organizations should exploit this phenomenon in order to improve recruitment and retention of volunteers. Specifically, EMS organizations can establish partnerships with employers of OGS, which can lead to higher participation rates. For example, MDA works in collaboration with the Israeli Defense Forces Medical Corps in an effort to encourage army medics, paramedics, and nurses to join the Life Guardians.

***Social*,** surprisingly, was found to be significantly higher in OGS than in TDV in spite of the fact that OGS have no regular meetings or social activities like TDV. On the other hand, the relative importance of this function is as expected as *social* ranked highest in importance for TDV and only third in importance for OGS. The magnitude of the difference is low at 0.68 points or 18% (compared to an average magnitude of 1 point or 34%). Further research is needed in order to investigate the causes of this unexpected difference.

***Protective*** is significantly higher in OGS than in TDV. This function ranked second in importance for OGS and third in importance for TDV. The magnitude of the difference is relatively high at 1.26 points or 36%. The implication of this finding is that protective-oriented activities designated for recruitment and retention of volunteers can be more effective for OGS than for TDV.

Most results of the binary logistic regression are consistent with the results of other techniques applied and complement them. Specifically, the probability of becoming a TDV was higher for individuals with higher *enhancement* and *values* volunteering functions. The lower importance of the *enhancement* volunteering function for OGS can be explained by the fact that OGS are older and many of them are certified medical professionals. While other techniques didn’t reveal a significant difference in the *values* volunteering function, the binary logistic regression suggests that the *values* volunteering function is more important for TDV than OGS.

The results of the J48 classification tree analysis are consistent with our previous findings and thus strengthen them.

### Limitations

This research examines one national EMS. Cultural differences can lead to different results in other countries, as observed by Anheier and Salamon [81]. Multinational research may be useful to explore how cultural differences influence the volunteering functions of different groups of volunteers.

This study is limited to factors related to volunteer motivation, but does not address the effectiveness of different types of volunteers in emergency situations. The effectiveness of OGS should be studied along two dimensions: response time, i.e., in what percentage of cases do OGS arrive significantly earlier than the ambulance, and medical outcome, i.e., how does intervention by OGS influence survival rates and prognosis.

## Conclusion

This study revealed that Organized Good Samaritans form a separate category of volunteers. They differ from Time-Donation Volunteers in demography, professional background, and volunteering functions.

In emergency response services, as in many fields, technological developments and social changes alter the ways organizations act and the way society responds. In the constant effort to provide faster response times and save lives, the Organized Good Samaritans approach has the potential to become an important layer in emergency response infrastructure, extending the organization outward, and augmenting traditional methods with provision of faster first aid by a volunteer with better skills than a random bystander. Smartphone-based Organized Good Samaritans platforms provide an opportunity to volunteer to those who cannot donate time every week, but still want to help. Organized Good Samaritans can transition to become time-donation volunteers. The Organized Good Samaritans approach may be relevant in additional emergency management organizations, such as firefighters, rescue units, and even law enforcement.

This research described and formalized the Organized Good Samaritans approach emerging from recent technological and sociological developments. The results of this study provide important insights into Emergency Medical Services administrators and should enhance their ability to retain existing volunteers and recruit new ones, by adjusting their methods for each group of volunteers. Emergency Medical Services administrators can use these findings when considering and planning to introduce the Organized Good Samaritans approach.

## Data Availability

The data used for this research can be obtained from the corresponding author by sending a request by e-mail.

## Appendix

**Table 6:** Survey instrument – Clary and Snyder’s volunteer functions inventory

1. Volunteering can help me to get my foot in the door at a place where I would like to work.	
2. My friends volunteer.	
3. I am concerned about those less fortunate than myself.	
4. People I’m close to want me to volunteer.	
5. Volunteering makes me feel important.	
6. People I know share an interest in community service.	
7. No matter how bad I’ve been feeling, volunteering helps me to forget about it.	
8. I am genuinely concerned about the particular group I am serving.	
9. By volunteering I feel less lonely.	
10. I can make new contacts that might help my business or career.	
11. Doing volunteer work relieves me of some of the guilt over being more fortunate than others.	
12. I can learn more about the cause for which I am working.	
13. Volunteering increases my self-esteem.	
14. Volunteering allows me to gain a new perspective on things.	
15. Volunteering allows me to explore different career options.	
16. I feel compassion toward people in need.	
17. Others with whom I am close place a high value on community service	
18. Volunteering lets me learn things through direct, hands-on experience.	
19. I feel it is important to help others.	
20. Volunteering helps me work through my own personal problems.	
21. Volunteering will help me to succeed in my chosen profession.	
22. I can do something for a cause that is important to me.	
23. Volunteering is an important activity to the people I know best.	
24. Volunteering is a good escape from my own troubles.	
25. I can learn how to deal with a variety of people.	
26. Volunteering makes me feel needed.	
27: Volunteering makes me feel better about myself.	
28. Volunteering experience will look good on my resume	
29. Volunteering is a way to make new friends	
30. I can explore my own strengths.	

## Abbreviations

EMS: Emergency Medical Services
OGS: Organized Good Samaritans
TDV: Time-Donation Volunteers
OHCA: Out-of-Hospital Cardiopulmonary Arrest
CPR: Cardiopulmonary Resuscitation
MDA: Magen David Adom (Israel National EMS)
AED: Automatic External Defibrillator
CFR: Community First Responder Group
EMT: Emergency Medical Technician
VFI: Volunteer Functions Inventory
RERN: Rural Emergency Responder Network
IDF: Israeli Defense forces
